# Assessing the Autoantibody levels in Relation to Recurrence of Pemphigus: Joint Modeling of Longitudinal Measurements and Recurrent Event Times

**DOI:** 10.5812/ircmj.13812

**Published:** 2014-02-03

**Authors:** Amal Saki Malehi, Ebrahim Hajizadeh, Kambiz Ahmadi, Parvin Mansouri

**Affiliations:** 1 Department of Biostatistics, Faculty of Medical Sciences, Tarbiat Modares University, Tehran, IR Iran; 2 Department of Biostatistics, Health Faculty, Jundishapur University of Medical Sciences, Ahvaz, IR Iran; 3 Skin and Stem Cell Research Center, Tehran University of Medical Sciences, Tehran, IR Iran

**Keywords:** Pemphigus, Recurrent Event, IgG Antibodies Titer, Joint Modeling

## Abstract

**Background:**

Pemphigus is an autoimmune bullous disease and it is unclear what triggers and deteriorates it. The current study aimed to evaluate whether increasing the IgG antibody titer represents a good indicator of the pemphigus recurrence.

**Objectives:**

The current study aimed to evaluate whether increasing IgG titer is an indicator of the expected recurrence.

**Patients and Methods:**

The current study was conducted at the Department of Dermatology, Imam Khomeini Hospital, Tehran University of Medical Sciences, between March 2007 and December 2012. A total of 112 patients with confirmed diagnosis of pemphigus based on clinical, histological and immuno-histological criteria were engaged in the study. The primary outcomes of the study were recurrent event times and IgG (Immunoglobulin G) antibody titer at each attendance. Joint model with shared random-effects was applied to assess the association between the two processes and investigate the affective factors.

**Results:**

Up to 8 recurrences were observed during the study time, but only 10% of the patients experienced more than 5 recurrences. A significant linear increasing trend in IgG antibody titer over time was found, IgG antibody titer increased 2.43% each month (P < 0.0001). The results showed positive correlation between IgG antibody titer and recurrence of pemphigus (P < 0.0001).

**Conclusions:**

The patients with higher IgG antibody titer were more likely to experience pemphigus recurrence. Therefore it can be concluded that titer of IgG and its increase may provide information regarding the progression of the pemphigus and the hazard of its recurrence.

## 1. Background

Pemphigus is a group of chronic skin disease and a life-threatening autoimmune disorder. It occurs when the body’s immune system attacks healthy cells. It causes blisters of the skin and mucous membranes. There are two main subtypes of pemphigus: pemphigus vulgaris and pemphigus foliaceus (1). The subtype of pemphigus depends on the blisters location. Most of the patients with pemphigus vulgaris have cutaneous blisters and erosions (2). pemphigus vulgaris is the most common form and can occur at any age, but often strikes people in middle age or older (2, 3). Pemphigus is characterized by loss of cell-cell adhesion (acantholysis) structures between epidermal keratinocytes due to binding of auto-antibodies to desmosomal adhesion structures. The Binding of auto-antibodies leads to the destruction of desmosomes, and thus to formation of the epidermal blisters or erosions (4, 5). The disease is generally considered to stem from a genetic predisposition, and triggered and exacerbated by one or more exogenous factors (6, 7). IgG, an antibody immune measure, is a well-known biomarker for pemphigus (8). Systemic corticosteroids and immunosuppressive drugs are usually used to treat pemphigus (5, 8). Despite the treatment, often clinical recurrence of disease may occur after a period of time. The estimated incidence of this disease is 1 per 100,000 of the population per year in Iran, which is relatively high compared with other countries (5). In addition, the risk for an aggravation or an abrupt recurrence of this autoimmune disease is very high (7, 9). In spite of the high incidence and recurrence rate of pemphigus vulgaris in Iran, the etiological studies in this field are very sparse. Since the pattern of IgG antibody titer is an important aspect of disease progression, it was monitored regularly. It might be determining how the risk of the recurrent event is influenced by the biomarker.

However IgG antibody titer was measured repeatedly over time and to assess the relationships between IgG antibody titer and pemphigus recurrence, joint modeling is a popular statistical analysis method. Joint modeling is usually used to investigate the relationship between both longitudinal measurements and event time data (10).

## 2. Objectives

The aim of this study is to evaluate whether increasing IgG titer is an indicator of expected recurrence.

## 3. Patients and Methods

The research was conducted as a longitudinal study at dermatology department of Imam Khomeini Hospital of Tehran from March 2007 to December 2012. Only those patients who regularly referred to the hospital were included. The diagnosis of disease was confirmed based on clinical, histological and immuno-histological criteria. After a preliminary assessment, a total of 102 patients were included in the study. The study sample was selected by simple random sampling technique. The primary outcomes of the study were recurrent event times and IgG antibody titer as biomarkers. Indirect immune-fluorescence (IIF) was used to detect serum auto antibody IgG for the diagnosis of the pemphigus and to evaluate the disease activity. Patients' information such as the demographic characteristics including age at diagnosis, gender, marital status and familial history used in the analysis were obtained from the hospital records. Other required data such as clinicopathological characteristics measured at a pathobiology laboratory on each visit , were also recorded. The current study was approved by the Ethical Committee of the Medical Science Faculty of Tarbiat Modares University, on August 20, 2012.

### 3.1. Data Analysis

In the current study the joint model with shared random-effects method was applied. In many medical studies both survival data and longitudinal biomarkers are observed together. For example, in this study, patients` antibody (e.g. IgG titers) was collected intermittently over time and the recurrence time of a pemphigus was also a matter of interest. The time of recurrence may be associated with the longitudinal biomarker trajectories. Separate analyses of longitudinal data and survival data may lead to biased results. Joint models incorporate all information simultaneously and provide valid and efficient inferences (11). The primary aim of joint analysis might be assessing how the changing of biomarker with time influences the risk of the events process. Joint analysis method models both longitudinal and survival processes simultaneously by linking them, and using unobserved random effects through the use of a shared parameter model. Shared random effect is the underlying framework for the joint modeling of longitudinal and event processes time (12). In other words random effect was applied to generate an association between the two processes where the longitudinal and recurrent event processes were assumed to be conditionally independent of given unobserved random effects. In the joint modeling, the linear mixed model was applied for modeling the longitudinal IgG antibody titer, and the Cox proportional hazard frailty model was applied for modeling the recurrent events. The IgG antibody titer was skewed therefore it was transformed to logarithms. Since many of the IgG antibody titers were 0, the value of 1 was added to all IgG titers, and then normal logarithms were taken. The model for the transformed IgG values was as follows:

Y_ij_ = X_i_β + α + e_ij_

**Figure 1.**
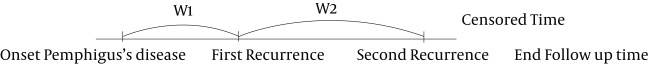
Schematic Pattern of Recurrent Event of Pemphigus Over Time and Gap Times Between the Sequential Events

Where α denotes a vector of random effects and it is independent of e_ij_ ~ N (0, σ^2^I). The current study used gap times between successive recurrences as the timescale for modeling recurrent events. Gap times were measured monthly and computed as W_ik_= T_ik_ – T_ik-1_ which i is indices of patients and k is indices of recurrent event number (Figure 1). Time of the disease onset was considered T_i0_=0 and corresponded to the start of the event process. The model for the recurrent events process is:

h_i_ (w) = h_0_ (w) exp (Z_i_β + γα)

Where it is assumed to be normal and determines the association between the IgG antibody titer and recurrent event process.

Baseline covariates including the joint model are: gender (1:male, 0:female), marital status (1:single, 0:married), clinical phenotype (1:skin lesions, 0:mucosal lesions), age and lymphocyte values. We are also interested in the rate of change of IgG over time, therefore the time since onset of pemphigus disease in months was included in the model for longitudinal measures as a covariate. 

The joint modeling was implemented by Proc NLMIXED in SAS version 9.1.

## 4. Results

**Figure 2.**
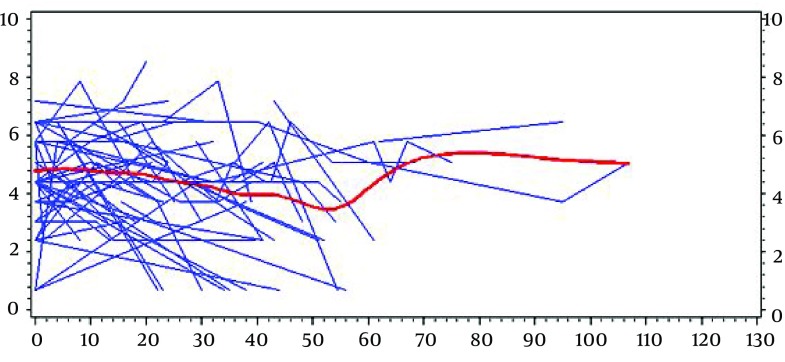
Individual Profile With Average Trend Line of IgG Over Time

**Figure 3.**
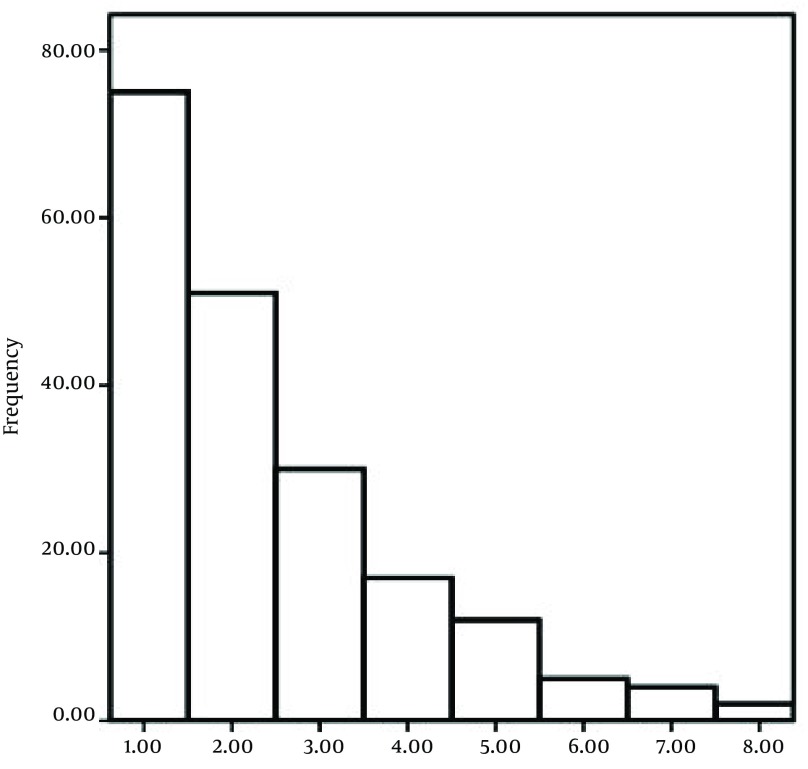
Number of Recurrent Pemphigus

A total of 112 patients who had been diagnosed with pemphigus were studied. The mean ± SD age at diagnosis in year was 47.5 ± 15.98 and the mean of follow-up time was 39.1 months. The demographical characteristics of the patients are presented in Table 1. The pattern of IgG values over time and the number of recurrences of the diseases are shown by exploratory plots in Figures 2 and 3. The estimated results using the joint model are presented in Table 2. Based on the obtained result, there was no significant difference between male and female (HR = 0.895, P = 0.65) and different phenotype of pemphigus (HR = 0.927, P = 0.45) in hazards of recurrence in the joint model. Single patients had a little more hazards of recurrence than married patients (HR = 1.44, P = 0.32) but it was not significant. The results indicated a significant linear increasing trend in log (IgG + 1) values over time, actually IgG values increased 2.43% each month (P < 0.0001). Age, gender and lymphocyte values were associated with IgG values. Male patients had a higher mean of IgG values (P = 0.0003). Higher age and lymphocyte level at baseline is associated with higher IgG values over time (P < 0.05). In addition, for the model association, the significant γ revealed a positive correlation between IgG antibody titer and pemphigus recurrence (P < 0.0001), which means that the disease is more likely to recur in patients with higher IgG antibody titer.

**Table 1.** The Demographical Characteristics of the Patients

Variable	Patients, (n = 112)	
	Mean ± SD	No. (%)
**Gender**		
Male	49.1 ± 13.48	67 (59.8)
Female	45.2 ± 18.88	45 (40.2)
**Marriage**		
Married		101 (90.2)
Single		11 (9.8)

**Table 2.** Result of the Joint Model of Recurrent Events and Longitudinal IgG

Covariate	Estimate	SE	P value	CI, 95%	
				Lower	Upper
**Longitudinal measures of IgG**					
Age ^a^	0.033	0.009	0.0008	0.014	0.051
Male ^a^	1.329	0.353	0.0003	0.629	2.029
Single	0.379	0.476	0.43	-0.567	1.33
Lymphocyte ^a^	0.041	0.015	0.009	0.011	0.072
Time ^a^	0.024	0.010	0.026	0.003	0.044
**Recurrent pemphigus event**					
Age	0.106	0.417	0.83	-0.711	0.923
Male	-0.111	0.244	0.65	-0.597	0.374
Single	0.367	0.369	0.32	-0.365	1.099
**Phenotype (skin lesions vs mucosal lesions)**	-0.076	0.101	0.45	-0.277	0.125
**γ ** ^**b**^	0.876	0.274	0.001	0.34	1.41

^a^ Statistically significant at 0.05 level.

^b^ Association parameter to indicate the correlation between the two outcomes.

## 5. Discussion

Recent reports revealed a high rate of pemphigus incidence in Iran (5, 13). In addition, high recurrence rate of pemphigus makes it one of the important concerns of dermatology (9). Therefore the current study was conducted to evaluate the prognostic factors of pemphigus recurrence and its association with pattern of IgG antibody titer. The present study utilized a new statistical methodology, joint modeling of survival and longitudinal biomarker data, to examine whether changes in IgG over time was associated with recurrence in pemphigus patients. The study showed no significant prognostic factor related to hazard of pemphigus recurrence. But single patients had a 44% more hazards of recurrence than the married ones (HR=1.44, P =0.32). However the findings of the current study disclosed that the values of IgG, and their increase can inform about the progression of pemphigus and the hazard of its recurrence. In general, there is a consensus between most of dermatological studies about role of the IgG antibody titer in clinical outcome of pemphigus (3, 14). The IgG, an antibody immune measure, is a well-known biomarker for pemphigus, and its pattern after treatment is an important aspect of the disease progression. Thus the longitudinal IgG values could be used to predict the recurrence in patients with pemphigus. This is an interesting finding, since it has long been conjectured that decreased antibody response is perhaps associated with longer relapse-free survival in pemphigus patients but there hasn't been any studies to prove it. Therefore, it is more important to monitor the outcome of the treatments by measuring the IgG antibody titer regularly. The main advantage of the current study was that in addition to evaluating the dependence between IgG and recurrent event processes, allowed us to investigate them through the joint model simultaneously. Therefore, in the current study, the pattern of IgG can be understood better. Based on the obtained results, male sex and age were positively associated with IgG values. The scarce studies focused upon the possible influences of these factors on the IgG values. The current study findings about gender are in general agreement with some, but not all, published data (15, 16). Numerous reports have indicated the statistically significant relationship between the levels of IgG and age (15-17). Furthermore results of the current study showed that baseline lymphocyte was associated with pattern and values of IgG, since subset of B lymphocytes had IgG on their surfaces. This induced the positive association between the lymphocytes and IgG levels. However, it should be mentioned that there is overwhelming consensus among all the studies that the genetic factor is the key factor of the onset and course of pemphigus, but is not sufficient to initiate the pemphigus (7, 18). Evaluating genetic factor needed expensive genetic testing which both the researchers and the patients could not afford, therefore there was one aspect of the disease that was not assessed in this study to investigate the claim. Just the family history was evaluated as a genetic indicator, but none of the patients had a positive family history.

The joint model was applied to investigate the IgG and recurrent processes simultaneously. The key result of this study demonstrated that the IgG pattern is suggesting an increased risk of clinical recurrence. Furthermore IgG has slow rate of increase over time (2.43% per month). Therefore it is very important to monitor the outcome of the treatments by measuring IgG antibody titer regularly. Observing an increase in the IgG titer, the clinicians may initiate to change treatment before the patient actually experiences a clinical recurrence. However, measuring IgG antibody titer can be helpful for clinicians in disease management and predicting prognosis.
